# Supplementary figures and images for: The effects of the COVID-19 pandemic on Italian primary school children’s learning: A systematic review through a psycho-social lens
Source: PLoS One. 2024 Jun 14;19(6):e0303991. doi: 10.1371/journal.pone.0303991 (PMC11178219; doi:10.1371/journal.pone.0303991)

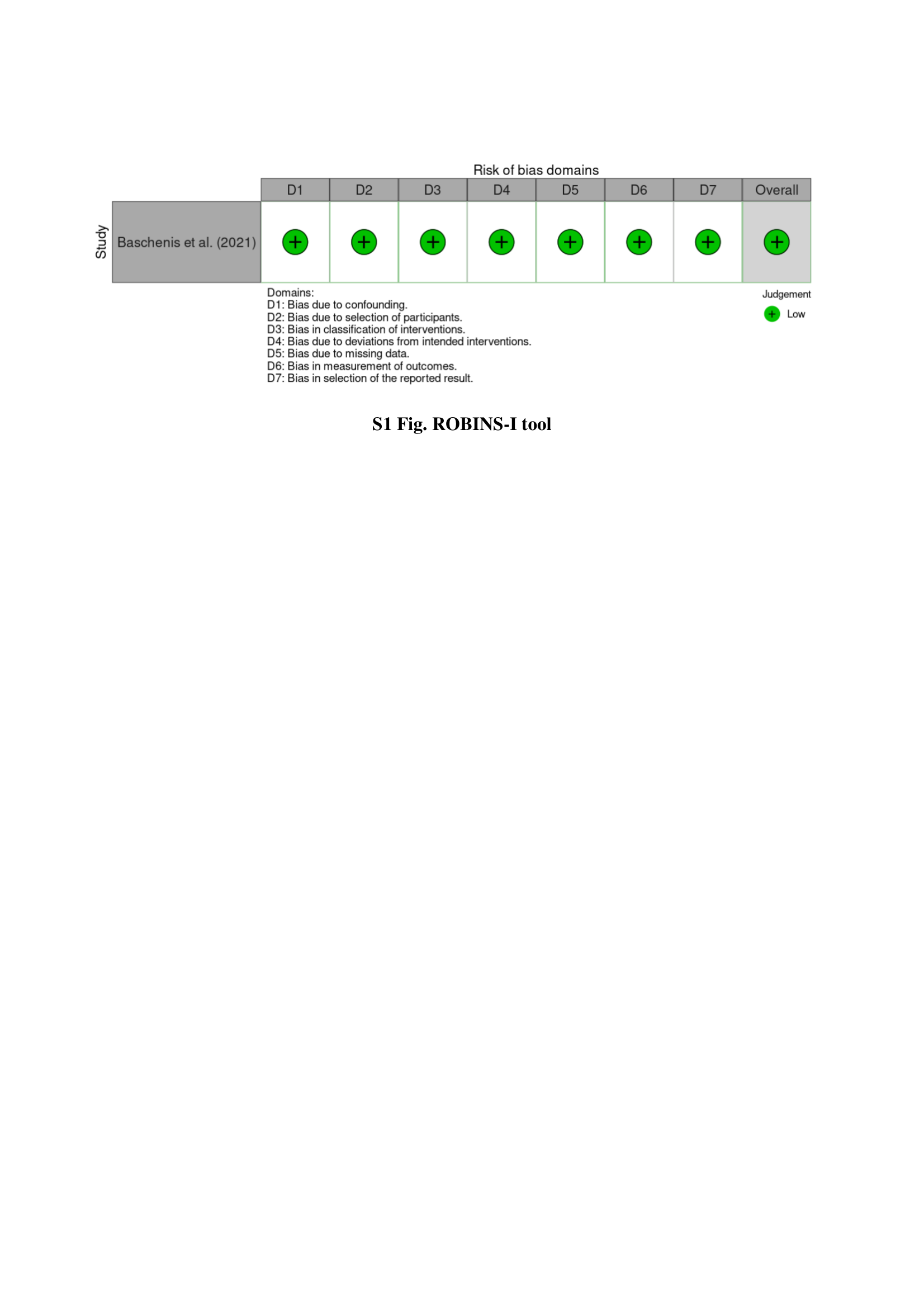

Supplement: S1 Fig — (TIFF) [file pone.0303991.s002.tiff]
