# Supplementary material for: The effects of the COVID-19 pandemic on Italian primary school children’s learning: A systematic review through a psycho-social lens
Source: PLoS One. 2024 Jun 14;19(6):e0303991. doi: 10.1371/journal.pone.0303991 (PMC11178219; doi:10.1371/journal.pone.0303991)
Supplement: S1 Table — (PDF) [file pone.0303991.s003.pdf]

Cohort studies (n=3)	Selection				Comparability	Outcome			Total (0-9) *
	Representativeness of the exposed sample	*Selection of the non-exposed cohort*	*Ascertainment of exposure*	*Demonstration that outcome of interest was not present at start of study*	*Comparability of cohorts on the basis of the design or analysis controlled for confounders*	*Assessment of outcome*	*Was follow-up long enough for outcomes to occur?*	*Adequacy of follow-up of cohorts*	
Bazoli et al. (2022)	1	1	1	1	1	0	1	1	7
Borgonovi & Ferrara (2023)	1	1	1	1	1	0	1	1	7
Contini et al. (2022)	1	1	1	1	1	0	1	1	7

S1 Table. Newcastle-Ottawa scale adapted for Cohort studies (NOS-C). * 9 = Very Good; 7–8 = Good; 5–6 = Satisfactory; 0– 4 = Unsatisfactory.
