# Supplementary material for: The effects of the COVID-19 pandemic on Italian primary school children’s learning: A systematic review through a psycho-social lens
Source: PLoS One. 2024 Jun 14;19(6):e0303991. doi: 10.1371/journal.pone.0303991 (PMC11178219; doi:10.1371/journal.pone.0303991)
Supplement: S2 Table — (PDF) [file pone.0303991.s004.pdf]

Case-control studies (n=1)	Selection				Comparability	Exposure			Total (0-9) *
	Is the case definition adequate?	*Representativeness of the cases*	*Selection of controls*	*Definition of controls*	*Comparability of cases and controls on the basis of the design or analysis*	*Ascertainment of exposure*	*Same method of ascertainment for cases and controls?*	*Non-response rate*	
Termine et al. (2022)	0	1	1	1	1	0	1	0	5

S2 Table. Newcastle-Ottawa scale adapted for Case-control studies (NOS-CC). * 9 = Very Good; 7–8 = Good; 5–6 = Satisfactory; 0– 4 = Unsatisfactory.
