# Supplementary material for: The effects of the COVID-19 pandemic on Italian primary school children’s learning: A systematic review through a psycho-social lens
Source: PLoS One. 2024 Jun 14;19(6):e0303991. doi: 10.1371/journal.pone.0303991 (PMC11178219; doi:10.1371/journal.pone.0303991)
Supplement: S3 Table — (PDF) [file pone.0303991.s005.pdf]

Cross-sectional studies (n=29)	Selection				Comparability	Outcome		Total (0-10) *
	Representativeness of the sample	*Sample size*	*Non-respondents*	*Ascertainment of the exposure*	*Adjustment of the outcome*	*Assessment of the outcome*	*Statistical test*	
Bazzoli et al. (2021)	0	0	0	0	0	1	1	2
Benigno et al. (2020)	0	0	0	0	0	0	0	0
Bertoletti et al. (2023)	1	1	0	2	1	0	1	6
Canals-Botines et al. (2021)	0	0	0	0	0	0	0	0
Capperucci et al. (2022)	0	0	0	0	0	0	0	0
Capurso & Roy Boco (2021)	0	0	0	0	0	0	0	0
Champeaux et al. (2022)	0	0	0	0	0	0	1	1
Colombo & Santagati (2022)	0	0	0	0	0	0	0	0
Crescenza et al. (2021)	0	0	0	0	0	0	0	0
Crisci et al. (2021)	1	0	1	2	0	0	1	5
Decarli et al. (2022)	0	0	0	0	0	0	1	1
Doz et al. (2022)	1	1	1	1	1	2	1	8
Ferretti, et al. (2021)	0	0	0	0	0	0	0	0
Gaggi et al. (2020, September)	0	0	0	1	0	0	0	1
Gentile et al. (2021)	0	0	0	0	1	0	1	2
Guzzo et al. (2022)	0	0	0	0	0	0	1	1
Ianes & Bellacicco (2020)	0	0	0	0	0	0	1	1
Inguscio et al. (2023)	0	0	0	2	0	0	1	3
Mangiavacchi et al. (2021)	0	0	0	0	0	0	1	1
Marchese et al. (2022)	0	0	0	0	0	0	0	0
Picca et al. (2021)	0	0	0	0	0	0	1	1
Ranieri et al. (2020)	0	0	0	0	0	0	0	0
Scarpellini et al. (2021)	0	0	0	0	0	0	1	1

**Cross-sectional studies (n=29)**	**Selection**				**Comparability**	**Outcome**		**Total (0-10) ***
	Representativeness of the sample	*Sample size*	*Non-respondents*	*Ascertainment of the exposure*	*Adjustment of the outcome*	*Assessment of the outcome*	*Statistical test*	
Segre et al. (2021)	0	0	0	0	0	0	1	1
Szpunar et al. (2021)	0	0	0	0	0	0	0	0
Termine et al. (2021)	0	0	0	2	0	0	1	3
Thorell et al. (2021)	0	0	0	0	0	0	1	1
Thorell et al. (2022)	0	0	0	2	0	0	1	3
Zaccoletti et al. (2020)	0	0	0	2	1	0	1	4

S3 Table. Newcastle-Ottawa scale adapted for Cross-sectional studies (NOS-CS). * 9 = Very Good; 7–8 = Good; 5–6 = Satisfactory; 0– 4 = Unsatisfactory.
